# Clinical parameters among patients in Japan with anemia and non-dialysis-dependent chronic kidney disease with and without diabetes mellitus who received roxadustat

**DOI:** 10.1007/s10157-022-02225-w

**Published:** 2022-04-24

**Authors:** Tadao Akizawa, Keiko Tanaka-Amino, Tetsuro Otsuka, Yusuke Yamaguchi

**Affiliations:** 1 Department of Nephrology, Showa University School of Medicine, Tokyo, Japan; 2 Medical Specialty, Japan Medical Affairs, Astellas Pharma, Inc., 2-5-1, Nihonbashi-Honcho, Chuo-ku, Tokyo, 103-8411 Japan; 3 Japan-Asia Clinical Development, Astellas Pharma, Inc., Tokyo, Japan; 4 Data Science, Development, Astellas Pharma, Inc., Tokyo, Japan

**Keywords:** Anemia, Chronic kidney disease, Diabetes mellitus, Roxadustat

## Abstract

**Background:**

Roxadustat is an oral hypoxia-inducible factor prolyl hydroxylase inhibitor for treating anemia of chronic kidney disease (CKD). This post hoc analysis of a Japanese, open-label, partially randomized, phase 3 study in patients with non-dialysis-dependent (NDD) CKD evaluated disease state–related parameters among patients with and without diabetes mellitus who received roxadustat. In the 1517-CL-0310 study (NCT02988973), roxadustat was noninferior to darbepoetin alfa for change in average hemoglobin levels at Weeks 18–24 from baseline who received roxadustat.

**Methods:**

Patients enrolled in the 1517-CL-0310 study who received roxadustat were included in this post hoc analysis. Hematologic (hemoglobin, reticulocyte/erythrocyte ratio, mean corpuscular volume [MCV], and mean corpuscular hemoglobin [MCH]), iron-related (ferritin, total iron-binding capacity, transferrin, ceruloplasmin, and hepcidin), metabolic (HbA1c, glycated albumin, total cholesterol, low-density lipoprotein cholesterol, and high-density lipoprotein cholesterol), and renal (eGFR) parameters were summarized descriptively by visit through Week 52.

**Results:**

Among 201 included patients, 105 (52.2%) and 96 (47.8%) were in the Diabetes and No Diabetes subgroups, respectively. There were no clinically meaningful differences through Week 52 for most hematologic, iron-related, metabolic, or renal parameters between patients in the Diabetes and No Diabetes subgroups. MCV and MCH remained lower and HbA1c and glycated albumin remained higher in patients in the Diabetes subgroup through Week 52. Both subgroups experienced a similar benefit from roxadustat in maintaining hemoglobin levels in the target range of 10–12 g/dL.

**Conclusion:**

Roxadustat maintained hemoglobin levels in the target range with similar clinical parameters irrespective of diabetes mellitus presence at baseline.

**Supplementary Information:**

The online version contains supplementary material available at 10.1007/s10157-022-02225-w.

## Introduction

The prevalence of anemia of chronic kidney disease (CKD) increases with declining renal function [1]. Additionally, the incidence and prevalence of diabetes mellitus and its negative sequelae are increasing worldwide, with a substantial share of this burden attributable to diabetic kidney disease (DKD) [2, 3]. Diabetes mellitus and progression of CKD are both associated with increased incidence of anemia [4]. Early identification and treatment of anemia are of paramount importance in DKD; however, treatment of anemia in patients with non-dialysis-dependent (NDD) CKD is often delayed or not initiated at all [5]. In Japan, erythropoiesis-stimulating agents (ESAs) are used in approximately 7.9% and 22.4% of patients with anemia and stage 4 and 5 CKD, respectively [5].

Reduced synthesis of erythropoietin, dysregulated oxygen sensing, functional iron deficiency, elevated hepcidin concentrations, and increased inflammation from disease states such as diabetes mellitus are characteristic of anemia of CKD pathogenesis [1, 6–8]. Anemia of CKD is associated with reduced health-related quality of life and elevated risk for hospitalization, mortality, and cardiovascular events as well as increased length of hospital stay [9, 10]. Additionally, increased rates of hospitalization and death have been observed in patients with diabetes mellitus and anemia of CKD [11].

Roxadustat, an oral hypoxia-inducible factor prolyl hydroxylase inhibitor, is approved in multiple countries, including Japan, for anemia in patients with dialysis-dependent (DD) and NDD CKD. Patients with NDD CKD receiving recombinant human erythropoietin (rHuEPO), darbepoetin alfa (DA), or epoetin beta pegol (EBP) were randomized or allocated to DA or roxadustat in a phase 3, multicenter, partially randomized, open-label study (1517-CL-0310) [12]. In this study, roxadustat maintained hemoglobin levels within the target range of 10–12 g/dL and was noninferior to DA while demonstrating a similar safety profile to previous studies in NDD CKD [13–16]. Because of the previously mentioned negative consequences of DKD and the potential impact roxadustat may have in this patient population, a post hoc subgroup analysis of the 1517-CL-0310 study was conducted to evaluate disease state–related parameters among patients with and without diabetes mellitus who received roxadustat.

## Materials and methods

### Study design

Patients enrolled in the open-label 1517-CL-0310 study (NCT02988973) receiving rHuEPO or DA before conversion were randomized to either the roxadustat (initial dose 70 mg or 100 mg three times weekly) or the DA (initial dose 10–60 µg every two weeks) treatment arm [12]. Patients receiving EBP before conversion were allocated to the roxadustat treatment arm (reference arm). Initial conversion doses were based on the prescribed ESA dose immediately prior to registration, and dose adjustments were made throughout the study according to prespecified criteria to maintain hemoglobin levels within 10–12 g/dL. Patients randomized or allocated to roxadustat were treated until Week 52 to evaluate long-term efficacy and safety; patients randomized to DA were treated until Week 24. Data through Week 52 from patients who received roxadustat were included in this post hoc analysis, whereas patients who received DA were excluded. Intravenous iron was restricted for use to maintain transferrin saturation ≥ 20% and/or serum ferritin ≥ 100 ng/mL at the discretion of investigators, and oral iron use was not allowed. This study was conducted in accordance with the ethical principles of the Declaration of Helsinki, Good Clinical Practice, the International Council for Harmonisation of Technical Requirements for Pharmaceuticals for Human Use guidelines, and applicable laws and regulations. The protocol was approved by each institutional review board (approval number 1517-CL-0310), and all subjects provided written informed consent.

### Study population

Eligible patients were aged ≥ 20 years; had a diagnosis of CKD (estimated glomerular filtration rate [eGFR] ≤ 89 mL/min/1.73 m^2^) but were not receiving dialysis; had anemia of CKD and had been receiving subcutaneous ESA, within the doses approved in Japan, for ≥ 8 weeks before prescreening assessments; and were considered to have stable hemoglobin levels, defined as 10–12 g/dL on the two most recent assessments. The full analysis set (FAS) for this post hoc analysis included patients who received ≥ 1 dose of roxadustat and who had ≥ 1 efficacy variable measured after the start of the study treatment, which totaled 201 patients.

### End points

The end points of this post hoc analysis were hematologic (hemoglobin, reticulocyte/erythrocyte ratio, mean corpuscular volume [MCV], and mean corpuscular hemoglobin [MCH]), iron-related (ferritin, total iron-binding capacity [TIBC], transferrin, ceruloplasmin, and hepcidin), metabolic (HbA1c, glycated albumin, total cholesterol, low-density lipoprotein cholesterol [LDL-C], and high-density lipoprotein cholesterol [HDL-C]), and renal (eGFR) parameters through Week 52. These parameters as well as patient demographics, baseline characteristics, and concomitant medication use through Week 52 were evaluated in patients with a diabetes mellitus diagnosis at baseline (Diabetes subgroup) and those without a diabetes mellitus diagnosis at baseline (No Diabetes subgroup).

### Statistical analysis

Demographic, baseline characteristics, and concomitant medication use were summarized descriptively. The hematologic, iron-related, metabolic, and renal parameters were also summarized descriptively by visit through Week 52. All analyses were conducted by diabetes mellitus diagnosis subgroup. All data processing, summarization, and analyses were performed using SAS^®^ v9.4 (SAS Institute Inc., Cary, NC).

## Results

### Patient disposition and baseline characteristics

Of the 201 patients included in this study, 105 (52.2%) were in the Diabetes subgroup and 96 (47.8%) were in the No Diabetes subgroup. In the FAS, most patient demographics and baseline characteristics were similar between the subgroups (Table 1). Patients were more commonly male (63.8% vs 53.1%) in the Diabetes subgroup, had a similar mean age between groups (68.8 vs 69.7 years), and had a lower BMI (24.48 vs 22.70 kg/m^2^) in the No Diabetes subgroup compared to patients in the Diabetes subgroup. The primary disease of CKD was more commonly chronic glomerulonephritis (34.4%), nephrosclerosis (43.8%), or other (21.9%) in the No Diabetes subgroup while most patients in the Diabetes subgroup developed CKD from diabetes (76.2%).

**Table 1:** Patient demographics and baseline characteristics (FAS)

Parameter	Diabetes (*n* = 105)	No diabetes (*n* = 96)
Sex (male), *n* (%)	67 (63.8)	51 (53.1)
Age, years	68.8 (10.7)	69.7 (11.8)
BMI, kg/m^2^	24.48 (4.42)	22.70 (3.95)
Primary disease of CKD, *n* (%)		
Chronic glomerular nephritis	12 (11.4)	33 (34.4)
Diabetes	80 (76.2)	0
Nephrosclerosis	13 (12.4)	42 (43.8)
Other	0	21 (21.9)
eGFR (mL/min/1.73 m^2^)	18.0 (8.8)	17.4 (6.9)
Baseline hemoglobin, g/dL	11.05 (0.58)	11.02 (0.54)
Ferritin (ng/mL)	143.63 (100.84)	141.52 (125.5)
Ferritin < 100 ng/mL, *n* (%)	43 (41.0)	44 (45.8)
Transferrin (g/L)	1.997 (0.344)	2.012 (0.384)
TIBC (µmol/L)	47.3 (7.2)	47.9 (7.7)
TSAT (%)	33.32 (11.62)	34.93 (12.27)
TSAT < 20%, *n* (%)	10 (9.5)	8 (8.3)
Soluble transferrin receptor (nmol/L)	24.11 (9.61)	22.78 (10.58)
Hepcidin (ng/mL)	40.36 (26.11)	38.21 (26.29)
Reticulocytes/erythrocytes	0.0111 (0.0053)	0.0109 (0.0057)
Erythrocyte MCV (fL)	91.49 (4.56)	94.59 (4.68)
Erythrocyte MCH (pg)	29.93 (1.77)	30.79 (1.63)
Erythrocyte MCH concentration (g/L)	327.1 (10.2)	325.6 (9.7)
Ceruloplasmin (mg/L)	256.4 (45.7)	261.3 (40.0)
Hemoglobin A1c (%)	6.52 (0.93)	5.64 (0.33)
Glycated albumin (%)	19.18 (3.55)	15.60 (1.68)
Total cholesterol (mmol/L)	4.578 (1.078)	4.746 (0.945)
LDL cholesterol (mmol/L)	2.608 (0.819)	2.648 (0.658)
HDL cholesterol (mmol/L)	1.368 (0.438)	1.478 (0.488)
Systolic blood pressure (mm Hg)	138.2 (19.8)	133.5 (17.4)
Diastolic blood pressure (mm Hg)	68.9 (11.5)	74.2 (12.4)

All values are presented as mean (standard deviation) unless otherwise specified

*BMI* body mass index, *CKD* chronic kidney disease, *FAS* full analysis set, *HDL* high-density lipoprotein, *LDL* low-density lipoprotein, *MCH* mean corpuscular hemoglobin, *MCV* mean corpuscular volume, *TIBC* total iron-binding capacity, *TSAT* transferrin saturation

### Concomitant medication use through 52 weeks

A numerically greater percentage of patients in the Diabetes subgroup compared to those in the No Diabetes subgroup were receiving oral iron (36.8% vs 28.6%), statin (50.0% vs 34.9%), anti-diabetic agent (69.1% vs 0) including insulin (39.7% vs 1.6%), or diuretic (58.8% vs 38.1%) through Week 52 (Online Resource 1).

### Clinical outcomes

#### Hematologic parameters

**Fig. 1:**
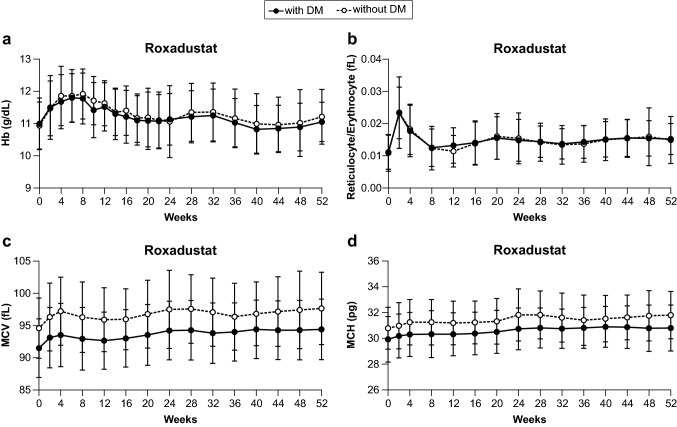
Hematologic parameters through Week 52. Black lines and markers and dotted lines with white markers denote mean (standard deviation) for Diabetes subgroup and No Diabetes subgroup patients, respectively. Individual figure panels demonstrate **a** hemoglobin, **b** reticulocyte/erythrocyte ratio, **c** mean corpuscular volume (MCV), and **d** mean corpuscular hemoglobin (MCH). *DM* diabetes mellitus, *Hb* hemoglobin

Hemoglobin levels in the Diabetes and No Diabetes subgroups initially increased before slowly decreasing and plateauing through Week 52 with mean values remaining between 10 and 12 g/dL (Fig. 1a). After an initial increase in the mean reticulocyte/erythrocyte ratio during the first 2 weeks in the Diabetes and No Diabetes subgroups, this ratio decreased but did not reach mean baseline values before stabilizing through Week 52 (Fig. 1b). Both the mean MCV and MCH were lower at baseline and through Week 52 for patients in the Diabetes subgroup compared to the No Diabetes subgroup, though the differences between baseline and through Week 52 were similar for both subgroups (Fig. 1c and d).

#### Iron-related parameters

**Fig. 2:**
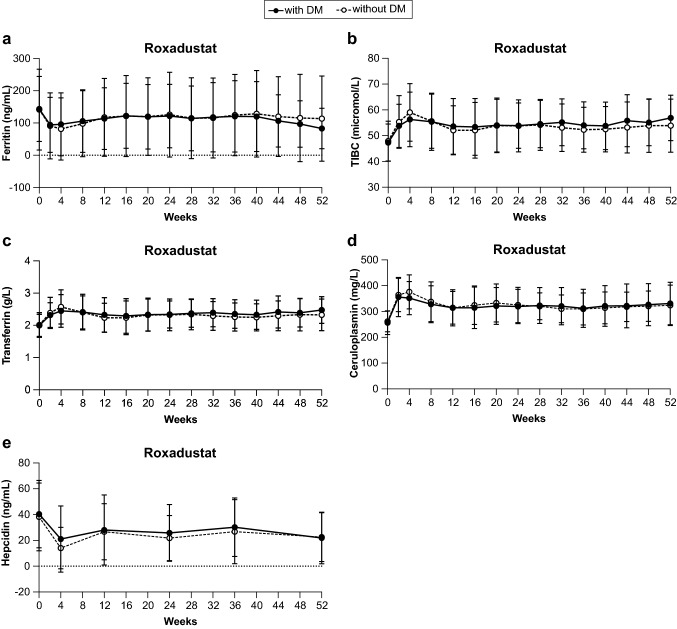
Iron-related parameters through Week 52. Black lines and markers and dotted lines with white markers denote mean (standard deviation) for the Diabetes subgroup and No Diabetes subgroup patients, respectively. Individual figure panels demonstrate **a** serum ferritin, **b** total iron-binding capacity (TIBC), **c** transferrin, **d** ceruloplasmin, and **e** hepcidin. *DM* diabetes mellitus

There were no differences in iron-related parameters through Week 52 between the Diabetes and No Diabetes subgroups. Mean serum ferritin decreased below both the baseline mean ferritin level and 100 ng/mL at Week 2 before stabilizing through Week 52 (Fig. 2a). Mean TIBC, transferrin, and ceruloplasmin each increased through Week 4 before decreasing and stabilizing at values higher than baseline through Week 52 (Fig. 2b–d). Mean hepcidin decreased approximately 50% to approximately 20 ng/mL at Week 4 before increasing and stabilizing below the baseline value through Week 52 in both subgroups (Fig. 2e).

#### Metabolic parameters

**Fig. 3:**
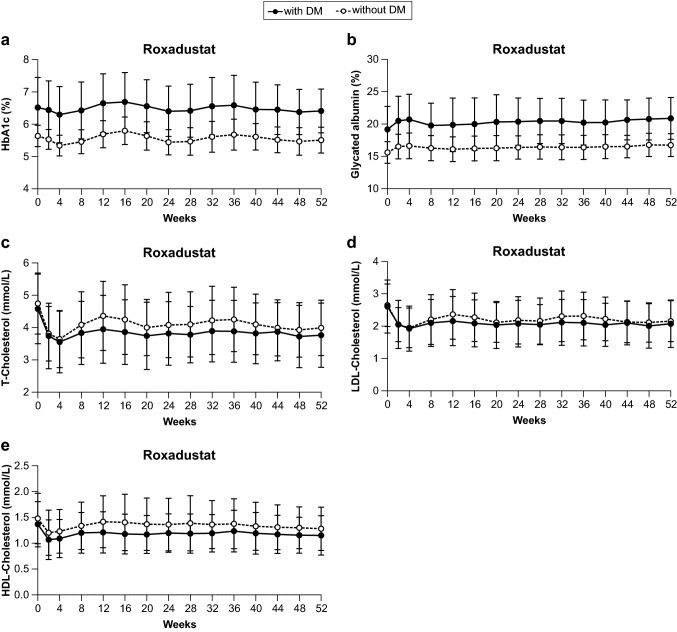
Metabolic parameters through Week 52. Black lines and markers and dotted lines with white markers denote mean (standard deviation) for the Diabetes subgroup and No Diabetes subgroup patients, respectively. Individual figure panels demonstrate **a** HbA1c, **b** glycated albumin, **c** total cholesterol, **d** low-density lipoprotein cholesterol (LDL-C), and **e** high-density lipoprotein cholesterol (HDL-C). *DM* diabetes mellitus

Mean HbA1c initially decreased through Week 4 in both subgroups before fluctuating between 6% and 7% in the Diabetes subgroup and between 5% and 6% in the No Diabetes subgroup through Week 52 (Fig. 3a). Glycated albumin initially increased through Week 4 in both subgroups before stabilizing at approximately 20% in the Diabetes subgroup and approximately 17% in the No Diabetes subgroup (Fig. 3b). Total cholesterol and LDL-C both initially decreased through Week 4 and HDL-C decreased through Week 2 before increasing and stabilizing through Week 52 though not reaching baseline values (Fig. 3c–e).

#### Renal parameter

Mean eGFR remained similar and stable between 15 and 20 mL/min/1.73 m^2^ through Week 52 in both subgroups (Online Resource 2).

## Discussion

This is the first analysis to evaluate changes in disease state–related parameters among patients with anemia with and without diabetes mellitus who received roxadustat. In patients with NDD CKD and anemia enrolled in this phase 3 study [12], there were no clinically meaningful differences in changes from baseline through Week 52 for most hematologic, iron-related, metabolic, or renal parameters between patients in the Diabetes subgroup and No Diabetes subgroup. MCV and MCH remained lower and HbA1c and glycated albumin remained higher in patients in the Diabetes subgroup through Week 52. Both subgroups maintained hemoglobin in the target range of 10–12 g/dL.

While the risk of anemia is greater in patients with, rather than without, diabetes [17], patients with diabetes treated with roxadustat experienced a similar initial increase in hemoglobin levels compared with patients without diabetes before plateauing to mean values between 10 and 12 g/dL through the end of this study; however, the dosage of roxadustat in each group was not evaluated and the comparative efficacy between patients with and without diabetes cannot necessarily be established from this post hoc analysis. Although no patients in either cohort had a competing diagnosis of microcytic anemia [18], the maintenance of target hemoglobin levels was achieved despite a lower baseline MCV and MCH in the Diabetes subgroup, which would be expected because of the pathophysiological effects of diabetes on these red blood cell parameters [19]. The red cell indices gradually increased in both subgroups through Week 52, suggesting improved quality of the red blood cells after roxadustat treatment that had not been observed in a prior 28-day study in patients in Japan [20, 21].

Iron-related parameters demonstrated considerable, but similar, changes in both the Diabetes and No Diabetes subgroups during roxadustat treatment. While serum ferritin initially decreased, TIBC, transferrin, and ceruloplasmin each initially increased. The decrease in serum ferritin may have been mediated by changes in the concentration of hepcidin, which can impair iron absorption from the duodenal enterocyte and iron release from macrophages to limit iron availability, especially in diseases with increased inflammation [1, 22]. Roxadustat patients with adequate iron repletion were observed to have the lowest required doses of roxadustat to maintain target hemoglobin levels in a prior post hoc analysis [23]. Roxadustat remains effective in the absence of the iron repletion requirement carried by ESAs [24–27], likely by promoting iron absorption and recycling from the macrophage iron storage system [28, 29].

While roxadustat had minimal or no effect on intermediate (glycated albumin) and long-term (HbA1c) markers of glycemic control, changes in cholesterol values were observed in the Diabetes and No Diabetes subgroups. Total cholesterol and LDL-C decreased initially and were maintained at levels lower than baseline through Week 52 irrespective of diabetes status, confirming prior findings in phase 2 and 3 studies [24, 25, 30]. The decrease in HDL-C is consistent with prior observations and occurs to such a small extent relative to LDL-C lowering that the LDL/HDL ratio is improved with roxadustat, as has been reported in other studies [31]. While 40–55% of patients were receiving a statin at Week 24, roxadustat likely contributes to these improvements as well irrespective of statin therapy use. In vitro studies have described the effects of hypoxia-inducible factor on acetyl coenzyme A that are required for the first step of cholesterol synthesis and in 3-hydroxy-3-methylglutaryl coenzyme A reductase degradation, the rate-limiting enzyme in cholesterol synthesis [32–34].

There were no considerable changes in eGFR in the Diabetes or No Diabetes subgroups through Week 52. A meaningful decrease in eGFR would not be expected over 52 weeks, thus the potential impact of roxadustat in either patient population on this parameter requires additional investigation [35]. Some of the worsening of anemia outcomes resulting from CKD progression comes from increased hepcidin concentrations, impaired iron metabolism, and decreased ESA efficacy, which might potentially be mitigated by roxadustat [36]. The effects on long-term outcomes from roxadustat in patients with NDD CKD who progress in their disease and require dialysis need further evaluation.

These study results should be considered hypothesis-generating as they were a post hoc analysis of the 1517-CL-0310 study and descriptive comparisons rather than inferential statistics were performed [12]. Most patient characteristics were similar between the Diabetes and No Diabetes subgroups, though some factors, such as medication use and the natural progression of diabetes and DKD, could have affected parameters regardless of roxadustat use. The Japanese patient population of this study may limit generalizability to other populations. In particular, parenteral iron use was restricted according to the study design, which may differ from treatment strategies used for many NDD CKD patients in other healthcare systems [37]. Because not all disease state–related parameters could be evaluated, select, clinically relevant parameters may be considered for future assessment. Additionally, there was no comparator agent evaluated in this analysis as patients randomized to DA received treatment through Week 24 rather than through Week 52 for patients who received roxadustat, which precluded comparison of longer-term outcomes of interest.

There were no clinically meaningful differences between patients with NDD CKD and anemia with or without diabetes mellitus enrolled in this phase 3 study for hematologic, iron-related, metabolic, or renal parameters. Roxadustat maintained hemoglobin levels in the target range regardless of diabetes diagnosis.

## Supplementary Information

Below is the link to the electronic supplementary material.

Supplementary file1 (DOCX 47 KB)

Supplementary file2 (PDF 406 KB)

## Data Availability

Researchers may request access to anonymized participant level data, trial level data and protocols from Astellas sponsored clinical trials at www.clinicalstudydatarequest.com. For the Astellas criteria on data sharing see: https://clinicalstudydatarequest.com/Study-Sponsors/Study-Sponsors-Astellas.aspx.
