# Supplementary material for: Clinical parameters among patients in Japan with anemia and non-dialysis-dependent chronic kidney disease with and without diabetes mellitus who received roxadustat
Source: Clin Exp Nephrol. 2022 Apr 24;26(9):843–50. doi: 10.1007/s10157-022-02225-w (PMC9385792; doi:10.1007/s10157-022-02225-w)

**Online Resource 1** Concomitant medication use through Week 52 (FAS)

| **Medication** | Diabetes  (n=68) | No Diabetes  (n=63) |
| --- | --- | --- |
| **Iron (oral)** | 25 (36.8) | 18 (28.6) |
| **Statin** | 34 (50.0) | 22 (34.9) |
| **Phosphate binder** | 5 (7.4) | 6 (9.5) |
| **Anti-diabetic agent^a^** | 47 (69.1) | 0 |
| **Insulin** | 27 (39.7) | 1 (1.6) |
| **Diuretic** | 40 (58.8) | 24 (38.1) |
| **Anti-hypertensive agent^b^** | 64 (94.1) | 60 (95.2) |

All values are presented as n (%)

^a^ Defined as alpha-glucosidase inhibitors, dipeptidyl peptidase-4 inhibitor, glucagon-like peptide-1 receptor agonist, peroxisome proliferator-activated receptor-gamma agonist, sodium glucose co-transporter 2 inhibitor, sulfonylurea

^b^ Defined as angiotensin-2 receptor antagonist, angiotensin-converting enzyme inhibitor, aldosterone antagonist, alpha-1 receptor antagonist, alpha-2 receptor agonist, beta blocker, calcium channel blocker, kallidinogenase

*FAS* full analysis set

**Online Resource 2** Estimated glomerular filtration rate (eGFR) through Week 52. Black lines and markers and gray lines and markers denote mean (standard deviation) for Diabetes subgroup and No Diabetes subgroup patients, respectively

*DM* diabetes mellitus


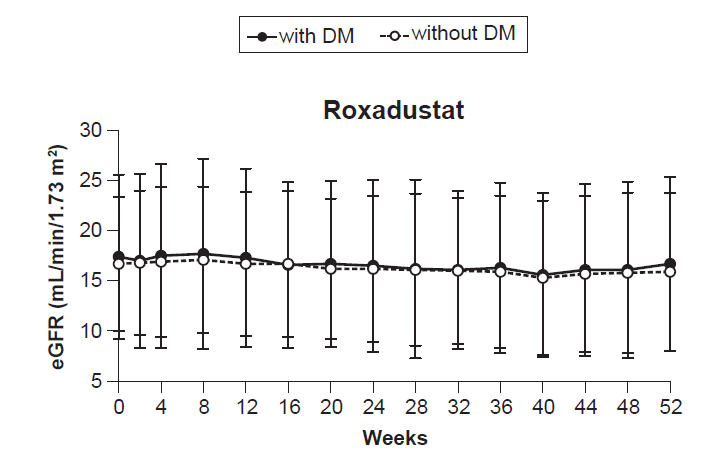

Supplement: Supplementary file 1 — Supplementary file1 (DOCX 47 KB) [file 10157_2022_2225_MOESM1_ESM.docx]
