# Supplementary figures and images for: Clinical parameters among patients in Japan with anemia and non-dialysis-dependent chronic kidney disease with and without diabetes mellitus who received roxadustat
Source: Clin Exp Nephrol. 2022 Apr 24;26(9):843–50. doi: 10.1007/s10157-022-02225-w (PMC9385792; doi:10.1007/s10157-022-02225-w)

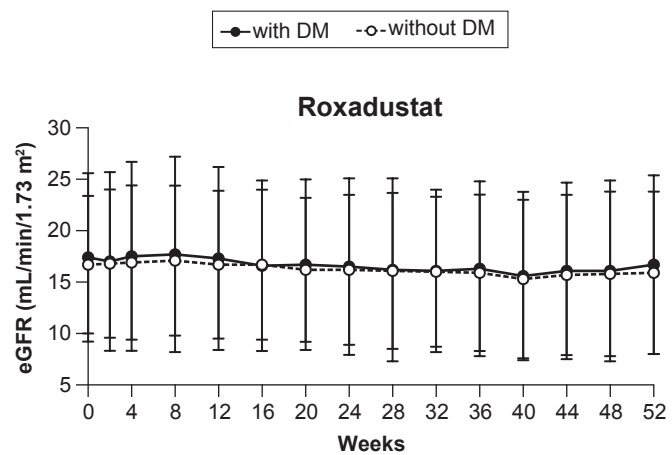

Supplement: Supplementary file 2 — Supplementary file2 (PDF 406 KB) [file 10157_2022_2225_MOESM2_ESM.pdf]
